# Medical Items

**Published:** 1914-03

**Authors:** 


### MEDICAL ITEMS

### COMMON SENSE IN MANAGING DIGESTIVE DISORDERS.

The artificial digestives have their place in modern therapy, but their use should always be tempered with sound judgment. Otherwise conditions we seek to correct are very apt to be aggravated. Undoubtedly we have placed too much reliance upon the digestives and as a consequence have often done more harm than good. As one physician states in referring to the digestive functions "Nature should be gently assisted in the human economy. If we persist in doing its work, like the lazy man, it soon depends on that assistance. This is true to the action of the liver, stomach, intestines, as well as all functions of the human anatomy. How often do we meet with individuals who are habitually dosing themselves whenever an action of the liver or bowels is required, and others who require a dose of pepsin or one of the many artificial digestives after each meal. This is all wrong. The functional activity of the entire process of digestion should be encouraged and any remedy that will specificially stimulate these functions to their healthy action is of greater service in digestion than pepsin. **Seng, a remedy that** has a well defined secernent action on the glands and mucous membranes of the stomach and intestines, thus compelling the secretion of Nature's own digestive fluids, is the modern treatment for dyspepsia,

### TO LIGHTEN THE HEART'S BURDEN.

In general practice few remedies are more commonly needed than a safe and reliable cardiac tonic. Not by any means a drug or remedy that will simply whip up and stimulate an organ that may already be laboring under a burden over heavy for its capacity, but on the contrary, a remedy that will impart new strength to the heart and by giving it new power and tone, relatively lighten the load it is carrying. Such a cardiac tonic is Cactina Pillets a remedy that in the hands of thousands of competent physicians has been found one of the most valuable in the whole realm of therapeutics. In countless cases of cardiac disease it has been employed with the most positive and gratifying results.

It is especially indicated in all functional heart diseases such as tachycardia, palpitation, feebleness; in the cardiac weakness and nervousness caused by excessive use of tobacco, tea, coffee or alcohol; to sustain the heart throughout many chronic and febrile diseases; to strengthen the heart of the convalescent; and for its support during the period of gestation. It has proven itself the safest cardiac tonic when the musculo-motor action of the heart requires strengthening or guarding and seems to promote the nutrition of the whole organ through its effect on the local circulation. In consequence there is ample justification for the statement so often made in describing Cactina Pillets that this remedy is a persuasive cardiac tonic, not a therapeutic lash.

